# Metabolic rate of angiosperm seeds: effects of allometry, phylogeny and bioclimate

**DOI:** 10.1098/rspb.2024.2683

**Published:** 2025-02-19

**Authors:** Emma L. Dalziell, Sean Tomlinson, David J. Merritt, Wolfgang Lewandrowski, Shane R. Turner, Philip C. Withers

**Affiliations:** ^1^ School of Biological Sciences, The University of Western Australia, Crawley, Western Australia 6009, Australia; ^2^ Kings Park Science, Department of Biodiversity, Conservation and Attractions, Western Australia, Kings Park, Western Australia 6005, Australia; ^3^ Biodiversity and Conservation Science, Department of Biodiversity, Conservation and Attractions Western Australia, Kensington, Western Australia 6151, Australia; ^4^ School of Biological Sciences, The University of Adelaide, Adelaide, South Australia 5000, Australia; ^5^ School of Molecular and Life Sciences, Curtin University, Bentley, Western Australia 6102, Australia

**Keywords:** seed mass, wild species, metabolic scaling, ecological correlates, residual analysis

## Abstract

Energetics is considered a fundamental ‘currency’ of ecology and the way that metabolic rate (MR)—the rate of energy expenditure on biological processes—scales relative to the size of the organism can be both an adaptive benefit and a constraint in mediating the energetic demands of ecological processes. Since few investigations have examined this relationship for angiosperm seeds, we measured standard metabolic rate (SMR) of 108 species’ seeds, spanning a broad suite of species. We used fluorescence-based closed-system respirometry at temperatures between 18°C and 30°C, based on optimal germination conditions, and Q_10_ corrected to 20°C. The allometric relationship for SMR as a function of seed mass was 0.081 × M^0.780^ with ordinary least squares regression and 0.057 × M^0.746^ with phylogenetic generalized least squares regression. This relationship is consistent with the pervasive metabolic allometry documented for both vegetative plants and domesticated cultivars (*n* = 14) which had higher SMR residuals than wild species (seven weeds and 87 Australian native species). For native species, seed SMR was strongly related to measures of increasing environmental aridity (annual mean temperature and precipitation, and precipitation in the wettest quarter), consistent with seeds from arid environments having a high MR to supply energy needed to germinate rapidly. By comparing SMR of seeds for diverse angiosperm species, we provide insights into inter-relationships of physiology, distribution, climate and domestication on seed ecology and suggest that energetics represents a valuable addition to established functional trait libraries for seed biology.

## 1. Introduction

Metabolic rate (MR), which is the rate at which an organism uses biochemical energy (typically adenosine tri-phosphate (ATP)) for its biological processes [1,2], is a core functional trait that underlies the processes that structure populations and ecosystems and can provide insight into the costs of living in diverse habitats and ecosystems [3–5]. MR is allometrically related to mass (*M*) and a broad suite of physiological and ecological processes across organisms [6–12]. Understanding these relationships enables us to make comparisons of maintenance energetic requirements across taxa and environments. The allometry of MR with mass is one of the most pervasive physiological relationships found across biological systems [13,14]. It is usually described by a power function MR = *aM^b^* where *a* is an intercept coefficient that varies between taxa, and *b* is the scaling slope of the relationship that is typically about 0.75 (i.e. allometric, not isometric) for both plants (including seeds) and animals [7,10,15,16]. Owing to this allometric relationship, larger organisms have a lower MR per unit mass in comparison with smaller organisms, meaning that larger and smaller organisms differ fundamentally in terms of their relative energetic requirements.

Despite the key role that seeds play in plant community ecology [17], there are few studies of the MR of seeds [18,19] with little consideration of variation between species or underlying associations with phylogeny, life history or environment. Rather, our understanding of seed metabolism has historically focussed on desiccation tolerance and controls of seed development and germination for species of agricultural importance [20,21] although there has been some notable work on wild species [22].

For seeds, mass spans approximately 12 orders of magnitude (approx. 0.001 mg to 20 kg [23,24]), so it is important to account for allometric effects when interpreting their MRs. It is equally important to account for the physiological state of the seeds during measurements of MR. Previous studies of seed metabolism have typically measured aerobic respiration when air-dried or moistened [18], or during the active phases of imbibition (water uptake) and germination as correlates of seed viability and vigour (e.g. for seed quality testing [21,25,26]). Seeds that have imbibed water and initiated germination have considerably higher MRs than quiescent, air-dry seeds that are metabolically depressed and anaerobic [26–28]. Metabolic activity of quiescent seeds varies with the level of hydration, and the threshold water potential for aerobic metabolism is *ca* 90−95% equilibrium relative humidity (equivalent to approx. −10 MPa [28–30]). Furthermore, temperature has an established effect on MRs [31], which is accounted for in the measurement of ectothermic animals by measuring MR at a standard temperature (e.g. 20°C), or at a thermal optimum. It is important for comparison of seeds of various species to use standard metabolic rate (SMR), which reflects the minimum metabolism of seeds in a relatively quiescent, but not metabolically depressed state, representing energy costs of self-maintenance [32]. Consequently, we consider the definition of SMR for plant seeds to be aerobic metabolism measured at a relative humidity of about 95%, where MR is not lowered by desiccation or elevated by germination [19,33], and at a temperature where germination potential is optimized. This optimum temperature is species specific and dependent upon the thermal performance norms of germination [34–37]. This is equivalent to the definition of SMR for ectothermic animals [2].

To compare MR for seeds (and other organisms) of differing mass, it is important to first determine the allometry of SMR and then compare the residual SMR (how high or low their SMR is relative to allometric predictions), rather than absolute SMR (ml h^−1^) or mass-specific SMR (ml g^−1^ h^−1^), which is confounded by mass. Species residuals are calculated as the vertical deviation above or below the allometrically predicted value from the ordinary least squares (OLS) regression. To interpret the allometric relationship between seed SMR and mass across multiple species, it is also essential to consider the potential effect of evolutionary relationships (e.g. phylogenetic generalized least squares (PGLS) regression) [38,39]. Closely related species (i.e. those that share a recent common ancestor) will have more similar traits than distantly related species, reflecting phylogenetic inertia, and are therefore not statistically independent. Consequently, to correctly determine the allometric slope for a group of organisms, it is important to account for phylogeny [40–43], and species residuals can then be calculated as the vertical deviation above or below the PGLS allometrically predicted relationship. Some species will have higher MRs (positive residuals) than the allometry/phylogeny predicts, and others will have lower MRs (negative residuals), providing insight into how much more or less metabolically active the species is than would be expected for its size and phylogeny. Such variation in residual SMRs often reflects the effects of other physiological, ecological or climatological variables [44], as well as individual differences and experimental error.

We hypothesize that phylogenetic inertia and various physiological (e.g. maximum germination success and germination speed), evolutionary (e.g. phylogenetic heritage) and environmental (e.g. climatic regions) attributes will contribute to variation in SMR of seeds, as is known for other seed traits [45]. Germination speed, for example, varies between ecosystems, with rapid germination common in arid regions and slower germination more common in mesic environments [46–48]. Germination speed is also an important trait selected in crop domestication, where fast and synchronous germination is desirable [49]. Metabolic activity has an established importance to processes of seed longevity and germination that in turn underpin plant recruitment [19,21], including patterns of dormancy and quiescence [50]. We explored correlates of SMR residuals, for seeds that had been elevated to a non-depressed metabolic state following equilibration at 95% relative humidity (RH), to account for allometric and phylogenetic relationships. Our simplest first expectation was that cultivars would have higher SMR residuals, evolved by selection for uniform, rapid germination. Secondly, we had complex expectations concerning climate. Conventional allometries of adult animals have found lower relative MRs in species from arid ecosystems [40,41,51]; however, seeds from warmer, climatically unpredictable (i.e. arid) environments tend to have higher germination rates [48,52], similar to more rapid metabolic and developmental rates of holometabolous animals [53,54], developing within a limited optimal climate. We hypothesize that, if SMR is related to rapid germination, then high SMR will be present in species from arid ecosystems as a contributing factor to recruitment rates during short windows of opportunity. By quantifying these responses, we aim to provide a better understanding of MRs across a broad range of taxa from contrasting climate zones.

In this study, we first examined the allometric relationship of SMR for seeds of 108 species from 24 angiosperm families, including diverse native species from across ecosystems of differing climatic conditions in Western Australia as well as domesticated crop cultivars and introduced weeds. We tested the following hypotheses: (i) seed SMR will scale allometrically with mass, with a scaling exponent of about 0.75; (ii) more closely related species will have more similar SMRs in comparison with more distantly related species (i.e. there is a strong phylogenetic signal for SMR); (iii) there will be differences in the SMR residuals from the PGLS allometric relationship (which are independent of phylogeny and mass) that correlate with total germination percentage and germination speed for native species; (iv) the SMR residuals of native species will be lower than domesticated cultivars; and (v) the SMR residuals of native species from cooler mesic biogeographic regions will be lower than those from warmer, more arid environments.

## 2. Methods

### (a) Seed collection and processing

We investigated the relationship between mass and SMR for seeds of 108 species, from 19 orders and 24 plant families (electronic supplementary material, table S1). These species are either native to Western Australia (‘native’; *n* = 87; nomenclature follows Western Australian Herbarium [55]), are weeds that have become naturalized (‘weed’; *n* = 7 [55]) or are agriculturally important crop species (‘crop’; *n* = 14; see ‘Plant Status’ in the electronic supplementary material, table S1). Seeds of all native and weed species were sourced from wild populations, while the crop seeds were sourced commercially or from the Western Australian Department of Primary Industries and Regional Development (see ‘Origin’ in the electronic supplementary material, table S1). For all wild-collected species, seeds were sampled at the local population level whereby collections were made from 30 to 50 individual plants, and the collection pooled. Seeds of crop species were similarly collected at a local population level, from many hundreds of individuals, and pooled. Seeds were stored at Kings Park and Botanic Garden (Perth, Western Australia) in a controlled environment facility at 15°C and 15% RH prior to the start of experimentation. Seeds were assessed individually via X-ray (Faxitron Specimen Radiography System MX-20 Cabinet, Tucson, Arizona) to ensure that they contained an intact, undamaged embryo, and where applicable also contained intact endosperm/perisperm. Seeds that were empty or showed obvious signs of damage (i.e. cracks in the endosperm, insect damage, etc.) were discarded, so that all seeds were 100% filled prior to measurement.

Seeds of several of our study species have physical dormancy (PY [56]) that prevents water uptake. PY was broken (to allow for hydration of seeds to 95% RH prior to SMR measurement) using previously established protocols for each species, which included submersion in hot water (*ca* 95°C), abrasion of the seed coat with a pneumatic seed scarifier (Mater PSS2000, USA) or nicking of the seed coat with a scalpel blade (electronic supplementary material, table S1 [48,57–60]). Seeds with other forms of dormancy [61] were not treated prior to SMR measurements.

All seed mass data represent dry mass. To determine seed dry mass, three replicates of between 10 and 50 seeds (depending on seed size) were placed on pre-weighed pieces of aluminium foil, then dried in an oven at 103°C for 17 h [62]; dried seed material was then weighed to 0.00001 g with an electronic balance (Shimadzu AUW220D, Australia).

### (b) Respirometric measurement of seed standard metabolic rate

We measured the SMR of seeds by repeated-measurement, fluorescence-based closed system respirometry using Q_2_ technology (ASTEC Global, The Netherlands) after Tomlinson *et al*. [33]. To reduce the effect of microbial contamination on SMR, seeds were initially sealed inside porous nylon mesh bags and soaked in a 2% (w/v) calcium hypochlorite solution with two drops of surfactant (Tween 80®, Hurst Scientific, Perth, Australia) under partial vacuum (−80 kPa for 10 min on/off/on for 30 min). Following surface sterilization, seeds were rinsed three times in autoclave-sterilized reverse-osmosis water. Bags containing seeds were then soaked in a biocide solution (2% solution of Plant Preservative Mixture; Plant Cell Technology, Washington, DC, USA) for 10 min, before being removed and dried in a sterile laminar flow cabinet. The water potential of seeds prior to SMR measurement was standardized by equilibrating seeds to 95% RH (−10 MPa) by suspending seeds in a nylon mesh bag over a non-saturated solution of lithium chloride (LiCl approx. 4.8 g per 100 ml H_2_O, made using autoclave-sterilized water [63]), in an air-tight box (NHP Fibox, NHP Electrical Engineering Products, Sydney, Australia) held at 20°C for 7–10 days. This ensured that resting aerobic metabolism was measured [27,30]. To ensure seeds had reached the target RH prior to measuring SMR, a small subsample of seeds from each species was removed from their bags contained within the box and tested for equilibrium RH (HygroPalm, Rotronic, Crawley, UK), then discarded.

Respirometry chambers were 500, 1500 or 1800 μl polypropylene tubes (Sarstedt, Mawson Lakes, Australia) depending on seed size; tubes were autoclave sterilized prior to use. For larger seeds, 5500 μl polyethylene terephthalate glycol tubes (not autoclave safe) were soaked in 80% ethanol for 1 h and dried in a sterile laminar flow cabinet. The threads of the Q_2_ caps containing the fluorometric dye were surface sterilized using a cotton-tip soaked in 80% ethanol, before being dried in the laminar-flow cabinet. Depending on seed size, 2–150 individual seeds were placed inside each chamber and 10 replicate chambers were measured for each species. A series of blank reference chambers containing 200 μl of water and ambient air were used as standards, in addition to the Q_2_’s own internal standards [33]. The measurement temperature of the Q_2_ was species-specific and ranged between 18°C and 30°C, based on known optimal temperatures for germination (electronic supplementary material, table S1 [48,58,64]). The partial pressure of oxygen (*p*O_2_) was measured for individual chambers containing seeds every 30 min for 1–7 days, depending on the rate of O_2_ consumption. Data were discarded once chamber *p*O_2_ fell below 0.8 of initial, to reduce the potential influence of hypoxia on both SMR and on measurement accuracy [33].

### (c) Seed germination and viability

To ensure that seed mass for SMR measurement represented living seeds, we subjected seeds to germination and cut-tests [62] at the end of SMR measurements. For seeds of some species, dormancy break treatments were applied based on previously established protocols for each species. Depending on the species and their unique dormancy break and germination requirements, seeds were placed in Petri dishes (Techno Plas, St Marys, Australia) containing sterilized water agar (0.7% w/v solution of agar in water), water agar with the addition of gibberellic acid (GA_3_), or water agar with the addition of karrikinolide (KAR_1_) [65,66] (electronic supplementary material, table S1). Petri dishes were wrapped three times with plastic film (GLAD® Wrap) to reduce water loss and placed in incubators at optimal germination temperatures under alternating light/dark conditions under a daily 12 h photoperiod of 30 μmol m^−2^ s^−1^, 400−700 nm, cool white, fluorescent light. Germination was defined by radicle emergence >2 mm and was scored every 3–4 days, for a total period of up to eight weeks. Total germination percentage (i.e. maximum germination success, *G*_max_) was calculated as the total per cent of seeds that germinated after the maximum germination time (up to eight weeks). Nonlinear regression modelling was used to determine germination speed, with cumulative germination data over time analysed by fitting three-parameter log-logistic functions available from the ‘*drc*’-package [67] in R, and time to 50% germination (*T*_50_) was calculated using the *ED*() function [67,68]. If seeds did not germinate during eight weeks incubation, a cut-test was undertaken whereby seeds were dissected in half lengthways and visually inspected [62]. Seeds that had non-viable embryos— characterized by a darkening of the embryonic tissues—were considered dead and their data were discarded from subsequent analyses.

### (d) Data handling

All data calculations and statistical analyses were performed using the *R* environment version 4.2.2 [69] and implemented with RStudio version 2022.07.2 [70]. Calculation of seed SMR (rate of oxygen consumed; *V̇*O_2_, μl O_2_ seed^−1^ h^−1^) followed Tomlinson *et al*. [33], including correction for evolved partial pressures of CO_2_ (*p*CO_2_) and H_2_O (*p*H_2_O), correction to standard temperature and pressure conditions (following Vleck [71]), and noise-reduction for the raw Q_2_ data. Owing to the low signal-to-noise ratio of the raw Q_2_ data, measurements of *p*O_2_ were smoothed by applying a three-measurement moving window average [72]. The *V̇*O_2_ was then calculated from the slope of the *p*O_2_-time regression. We then used a linear regression approach whereby any individual replicate was assumed to be erroneous and therefore considered an outlier and omitted if its slope fell outside two standard deviations from the global regression slope for each species [33]. This was necessary because microbial contamination, which typically elevated SMR, was often not visible on the surface of seeds during respirometry measurements.

There is a substantial literature on interspecific variation in the respiratory exchange ratio (RER), and it largely relates to the metabolic substrate being oxidized (i.e. carbohydrate, oils or proteins [1]). Clearly, with interspecific differences in oil and carbohydrate composition of seeds [73,74], there will be variation in RER, but specific data relating to this for the diversity of seeds that we studied are lacking. Instead, we assumed a RER of 1, implying that MR is dependent on the oxidation of carbohydrate, as the least erroneous route to take in the context of how few RER data are available for resting seeds.

We measured seed SMR at temperatures equivalent to the optimal germination conditions [48], as we considered it to be the most physiologically and ecologically relevant temperature (*T*_t_, see the electronic supplementary material, table S1 for temperatures at which SMR_t_ was measured). However, to compare between species measured at different temperatures, we standardized SMR to a common temperature (*T*_20_) of 20°C using the *Q*_10_ relationship, as SMR_20_ = SMR_t_ *Q*_10_^(*T*_20_-*T*_t_)/10^ assuming a *Q*_10_ of 2.5 [1]. This is the approach typically used in similar studies [10,41,75].

### (e) Phylogenetic tree

We constructed a species-level phylogeny for our 108 study species using Treegraph 2 [76] adopting branching patterns and divergence times from TimeTree (https://timetree.org/ [77]). The placement of families within the tree was also checked against Magallón *et al*. [78] and adjusted as necessary. For many of the Australian native taxa, particularly those genera for which we had numerous species (e.g. *Acacia*), divergence patterns and times were obtained from published phylogenies (see the electronic supplementary material, table S3). If no phylogenetic information was available for a species within a genus, then branching patterns and divergence times were estimated. In a few instances, polytomies were present, and to avoid this, divergence times were arbitrarily offset by 1 Myr. This did not have an impact on our phylogenetic analysis considering the total height of the tree (160.5 Myr).

### (f) Metabolic scaling and phylogeny

All seed (dry) mass and *V̇*O_2_ data were log_10_-transformed prior to analysis. OLS regression was used to examine the relationship between log_10_ mass and log_10_ SMR. Since the relative error of mass measurement is considerably lower than that of SMR measurement, OLS regression was used rather than major axis or standardized major axis regression [13,79]. A single-sample *t*‐test was then used to determine whether SMR scaled isometrically (i.e. regression slope = 1) or allometrically (slope ≠ 1) with seed mass. We also compared our results with other published seed MR data [18,19], with all datasets corrected to a common measurement temperature of 20°C using a *Q*_10_ of 2.5. We used analysis of covariance (ANCOVA) to compare studies by constructing a linear regression of MR and seed mass using ‘study’ as a covariate (representing data of Garwood & Lighton [18], Dalziell & Tomlinson [19] and the present study), and using a post-hoc pairwise comparison of studies using the package ‘*emmeans*’ [80].

The strength of a phylogenetic pattern was assessed for log_10_ mass and log_10_ SMR by Pagel’s *λ* and Blomberg’s *K* using ‘*Phytools*’ [81]. To account for phylogenetic relatedness, we regressed our data for log_10_ SMR against log_10_ mass by PGLS to fit a linear regression between the two variables, using the phylogeny to account for co-variance of species [82]. We used a restricted maximum likelihood approach using the *R* package ‘*Ape*’ [83], with Pagel’s lambda (*λ*) set at the ‘optimal’ value and the residual *R*^2^ (*R*^2^_resid_) calculated for the maximum likelihood model [84]. Comparisons between the OLS and PGLS models were made using Akaike information criterion (AIC) and comparisons of log-likelihood scores. PGLS comparison was not possible for data of Garwood & Lighton [18] as we could not obtain species identity for their SMR data, or the dataset of Dalziell & Tomlinson [19] because geographical location was unavailable for some species. We then calculated species SMR residuals from the PGLS regression for examination of possible physiological, ecological and climatic patterns, since these residuals reflect SMR independent of seed mass and phylogeny.

### (g) Correlates of standard metabolic rate residuals

First, we assessed whether there was any relationship for the physiological variables of maximum germination success (*G*_max_) and time to 50% germination (*T*_50_) with SMR residuals using linear regression models. Second, we assessed the potential influence of selective breeding by comparing SMR residuals of native species (‘wild’), weeds (‘wild’), and agricultural cultivars (‘domesticated’; see ‘Origin’, electronic supplementary material, table S1) with a linear regression model. Since we found that this relationship was significant, we excluded ‘domesticated’ species from further analysis. We excluded crop species from climatic pattern analyses (as they had a significantly higher SMR residual) and weed species (because there were insufficient collection details for many of these species; see ‘Plant status’, electronic supplementary material, table S1).

We determined the climate classification for each native species by plotting the coordinates of the species’ collection locations over a raster of the Köppen-Geiger climate map [85]. The 87 Western Australian native species were collected from four climatic regions. The majority were from the southwestern corner of Western Australia (‘Csa’; temperate with a dry, hot summer) or from the Pilbara bioregion in the northwestern corner of Western Australia (‘BWh’ region; arid, hot desert, electronic supplementary material, table S2). Six species were from the mid-west or Kimberley regions (‘BSh’ region; arid, hot steppe), and one species (*Androcalva perlaria*) was from the southern coast (BSk region; arid, cold steppe; this zone was removed from subsequent analysis as *n* = 1 species). We analysed these data in two ways: firstly by traditional analysis of variance (ANOVA) of SMR residuals, which assumes no order to the independent variable (Köppen-Geiger climate region), and secondly by *a priori* contrasts where the Köppen-Geiger climate regions were ranked by increasing aridity from Csa to BSh and finally BWh. The ANOVA was constructed as a linear model with a Gaussian error distribution using the *‘base’* statistical package in R. The *a priori* contrasts were developed as a custom contrast matrix using the *‘base’* statistical package in R, assuming a forward orthogonal order.

Finally, we aligned the collection locations for our native seeds with models of climatic data estimated for current climatic conditions (1950–2000) according to the ‘*Bioclim*’ algorithms (www.worldclim.org/bioclim [86,87]). We examined 15 climate variables that have previously been associated with patterns of MR for animals, and plant seed traits [8,40,88,89]. The Bioclim variables [90,91] included in the analysis were: mean annual temperature, mean diurnal temperature range, isothermality, temperature seasonality, annual temperature range, mean temperature of wettest quarter, mean temperature of driest quarter, mean temperature of warmest quarter, mean temperature of coldest quarter, annual precipitation, precipitation seasonality, precipitation of wettest quarter, precipitation of driest quarter, precipitation of warmest quarter and precipitation of coldest quarter. Although some of these variables were strongly co-correlated (see the electronic supplementary material), we included them all as they have potentially different ecological and ecophysiological effects, regardless of their correlations. The potential contribution of these climatic parameters to SMR residuals was evaluated by backward stepwise regression for the climatic variables, without interaction terms; insignificant parameters were removed from successive models. The remaining significant variables were then analysed for their ANOVA contributions using type III sums of squares (without interaction terms) using the ‘*car*’ package [92]. Climate data for each of the species analysed are included in the electronic supplementary material, table S2.

## 3. Results

Seed dry mass spanned four orders of magnitude, from 0.135 mg for seeds of *Calothamnus quadrifidus* to 526 mg for seeds of *Adansonia gregorii* (electronic supplementary material, table S1) for our 108 study species. Absolute SMR, *Q*_10_-corrected to 20°C, ranged from 0.00162 μl O_2_ seed^−1^ h^−1^ for 0.259 mg seeds of *Ficinia nodosa* to 40.1 μl O_2_ seed^−1^ h^−1^ for 144 mg seeds of *Cicer arietinum*.

### (a) Ordinary least squares allometry of standard metabolic rate

**Figure 1.**
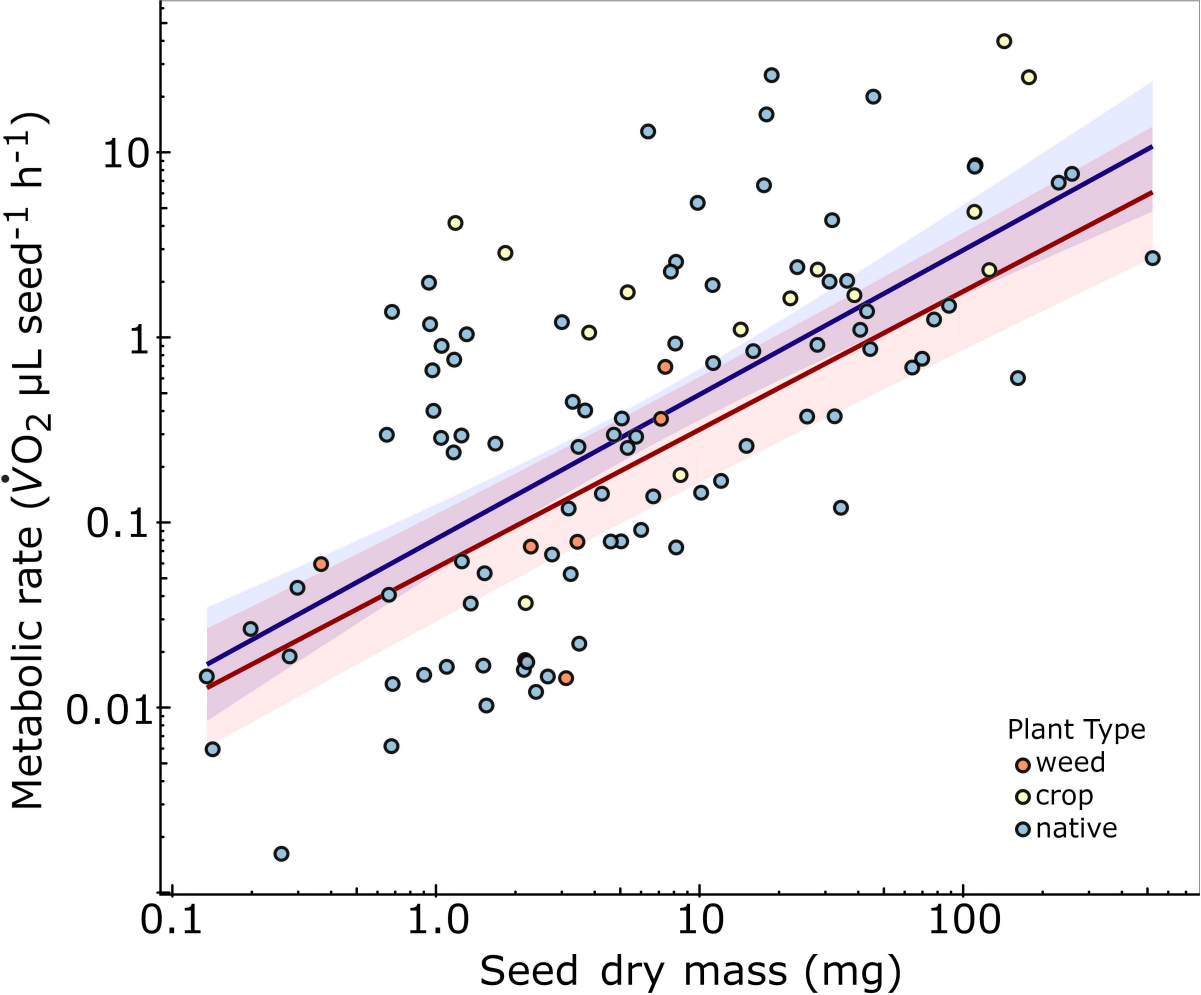
The allometric relationship between seed SMR (log_10_) and seed dry mass (log_10_) of 108 angiosperm species with ordinary least squares (OLS) regression (blue line) and phylogenetic least squares (PGLS) regression (red line). Shading represents the 95% confidence interval (CI) for each fitted regression. Species points are coloured according to the type of plant (weed, crop, native).

**Figure 2.**
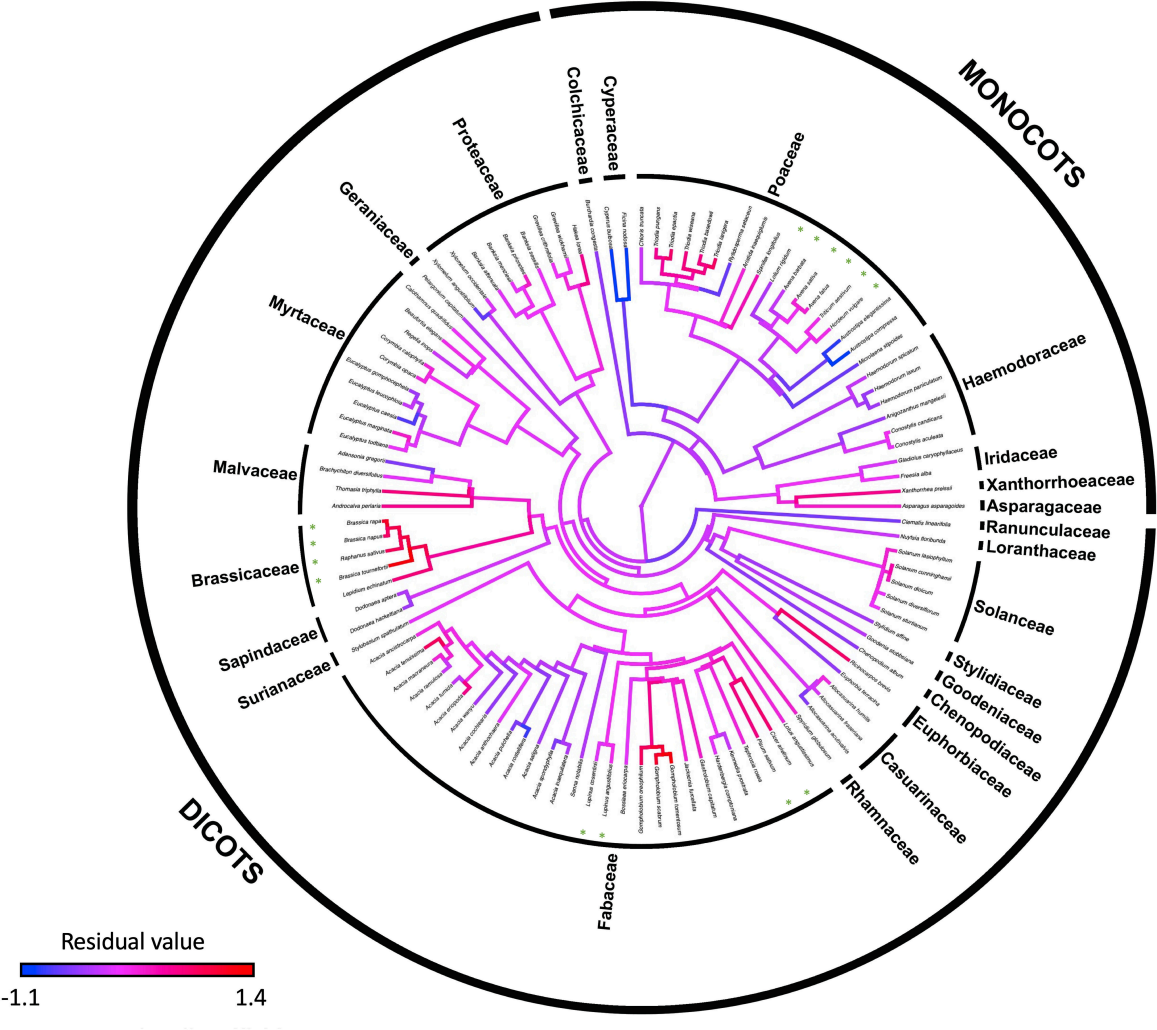
Phylogenetic tree for the 108 angiosperm species, showing residual log_10_ SMR values from OLS regression of log_10_ seed SMR against log_10_ seed mass; blue nodes indicate species that have lower SMR residuals, red nodes indicate species with higher SMR residuals. Domesticated species grown as crops are marked with green * (inside inner circle).

OLS regression of seed SMR (μL O_2_ h^-1^) had a positive relationship with seed (dry) mass; 0.0812 × *M*^0.780^ (*n* = 108 species; *F*_1,106_ = 85.2, *p* < 0.001, adjusted *R*^2^ = 0.440, AIC = 231; figure 1; table 1). A single-sample *t*‐test confirmed that this relationship was allometric (i.e. regression slope of 0.780 was significantly different from 1; *t*_106_ = 2.60, *p* = 0.011). There were SMR variations for genera within families and even species within genera (figure 2). For example, within Poaceae, seeds of *Triodia* had high, and *Austrostipa* had low, SMR residuals. There were no differences for SMR between prior datasets [18,19] and this study (*t*_129_ < 0.744, *p* > 0.738). For the combined dataset, seed SMR remained positively related to seed mass; 0.089 × *M*^0.797^ (*n* = 133 species, adjusted *R*^2^ = 0.521, *F*_1,131_ = 144.4, *p* < 0.001; electronic supplementary material, figure S1).

**Table 1.** Results of conventional (ordinary least squares (OLS)) regression and phylogenetically informed (phylogenetic generalized least squares (PGLS)) regression analyses for the allometric relationship between (log_10_) seed mass and (log_10_) seed SMR. (***p < 2.6 × 10^-16^.)

	parameter	estimate	s.e.	*t*	*R*^2^, AIC
conventional OLS	intercept	−1.09	0.094	*t*_106_ = 11.6***	*R*^2^ = 0.44 AIC = 231
	log mass	0.780	0.085	*t*_106_ = 9.23***	
phylogenetic PGLS	intercept	−1.24	0.289	*t*_106_ = 4.30***	*R*^2^ = 0.56 AIC = 232
	log mass Pagel’s *λ*	0.746 0.612	0.102	*t*_106_ = 7.32***	

### (b) Phylogenetic generalized least squares analysis of standard metabolic rate

There was a strong phylogenetic signal for both log_10_ mass (*λ* = 0.949 *p* < 0.001; *K* = 0.481, *p =* 0.001) and log_10_ SMR (*λ* = 0.906 *p* < 0.001; *K* = 0.394, *p =* 0.001). The optimal PGLS model (*λ* = 0.612) for seed SMR was positively related to mass; *V̇*O_2_ = 0.0570 × *M*^0.746^ (*n* = 108 species; AIC = 232.0; figures 1 and 2; table 1). The OLS and PGLS models for our data did not differ substantially (ΔAIC = 0.755; table 1; Burnham & Anderson [93]; loglikelihood ratio = 3.49, $\chi_{1}^{2}$ = 1.24, *p* = 0.264)

SMR residuals (μL O_2_ h^−1^) from PGLS regression, which account for variation in mass and phylogeny, showed no significant pattern with either maximum germination or germination speed (table 2). However, maximum germination was significantly higher (*t*_35_ = 4.04, *p* < 0.001) for crop species (87.8 ± 4.1, *n* = 14) than native and weed species (66.1 ± 3.4, *n* = 94), and germination time was significantly lower (*t*_30_ = 4.03, *p* < 0.001) for crop species (3.17 ± 0.96, *n* = 12) than native and weed species (8.27 ± 0.82, *n* = 65).

**Table 2.** ANOVA analysis for SMR residuals from PGLS regression. (For the maximum germination (*G*_max_)*,* germination speed (*T*_50_) and ‘crop’ vs ‘other’ comparison, factors were analysed separately by single-factor ANOVA; *n* = 86 for all other comparisons since crop and weed species were removed.)

factor	slope ± s.e.	*F,* d.f.	*p*	*n*
maximum germination (*G*_max_) %	+0.0039 ± 0.0020	*F*_1,106_ *=* 3.62	0.0597	108
germination speed (*T*_50_) days	−0.0188 ± 0.0122	*F*_1,75_ *=* 2.37	0.128	77
crop vs wild	+0.524 ± 0.192	*F*_1,106_ *=* 7.44	0.0075	108
Köppen-Geiger climate classification (*BSk removed)		*F*_2,83_ *=* 3.08	0.0512	86
ANOVA Csa, BSh, BWh *a priori* contrasts		*F*_1,84_ *=* 6.22	0.0146	86
annual mean temperature (°C)	+0.215 ± 0.067	*F*_1,79_ = 10.2	0.00204	86
annual precipitation (mm)	+0.0101 ± 0.0035	*F*_1,79_ = 8.49	0.00465	86
precipitation of wettest quarter (mm)	−0.0144 ± 0.0051	*F*_1,79_ = 7.89	0.00625	86
mean temperature diurnal range (mean of monthly (maximum temperature - minimum temp)) (°C)	−2.585 ± 1.289	*F*_1,79_ = 4.02	0.0484	86
annual temperature range (°C)	+1.257 ± 0.622	*F*_1,79_ = 4.08	0.0468	86
isothermality (%)	+73.36 ± 35.80	*F*_1,79_ = 4.20	0.0438	86

SMR residuals for crop species (0.638 ± 0.179) were significantly higher (*F*_1,106_ = 7.44, *p* = 0.007; table 2) than SMRs for wild (native and weed) species (−0.524 ± 0.192). The effect of Köppen-Geiger climate classification on SMR residuals for native species was marginally insignificant by ANOVA (*F*_2,83_ = 3.08, *p* = 0.051); however *a priori* contrasts, assuming an ordered increase of aridity between the Köppen-Geiger climate classifications, identified a significant effect of increasing aridity on SMR residuals (*F*_1,84_ = 6.22, *p* = 0.015). Backward stepwise regression indicated three highly significant climatic Bioclim variables (table 2), annual mean temperature (*p* = 0.0020), annual precipitation (*p* = 0.0047), and precipitation of wettest quarter (*p* = 0.0063); and three marginally significant Bioclim variables, isothermality (*p* = 0.0438), annual temperature range (*p* = 0.0458), and mean annual diurnal range (*p* = 0.0484).

## 4. Discussion

Our primary aims were first to establish the allometric and phylogenetic relationships between seed mass and SMR, and to then identify physiological, ecological and bioclimatic patterns in the residuals. For our dataset of 108 species, log_10_(SMR) was significantly related to log_10_(seed mass) by OLS regression with a slope of 0.78, or 0.83 when combined with two additional datasets (*n* = 133 [18,19]). There was a significant phylogenetic signal for seed mass (*λ* = 0.949) and SMR (*λ* = 0.906) (figure 2; table 1), and the optimal PGLS regression (with *λ* = 0.612), but the phylogenetically corrected allometric slope was similar at 0.75. These allometric slopes for SMR of seeds using OLS and PGLS regression are similar to the expected value of about 0.75 for both plants (including seeds) and animals [14–16,18,94] although near isometric scaling (i.e. *b* is about 1) has been reported for some plants [9,10,95] and seeds [19].

It is noteworthy that the OLS allometry of SMR with mass for seeds accounted for a significant but relatively modest proportion of the variance in our dataset (OLS *R*^2^ = 0.44). By contrast, other allometric OLS studies of SMR or basal metabolic rate (BMR) typically identify stronger patterns across plant (*R*^2^ = 0.98 [10]) and animal taxa, e.g.anuran amphibians (*R*^2^ = 0.86 [96]), reptiles (*R*^2^ = 0.96 [75]), mammals (*R*^2^ = 0.96 [11]), birds (*R*^2^ = 0.94 [12]) and insects (*R*^2^ = 0.82 [41]). Presumably, the higher variation in SMR residuals for seeds reflects a substantial contribution of factors other than mass to SMR, rather than just high measurement or individual errors. Three factors that we have identified as effectors of SMR are phylogenetic relationships, domestication and climate (see below), but further factors such as the proportion of metabolically active tissue (e.g. embryo size), seed dormancy (un-alleviated metabolic depression), and differences in metabolic substrate (e.g. oily vs starchy seeds), probably contribute variability to seed SMR residuals. Maternal effects, both genetic, and environmental, during seed development could also influence seed quality, e.g. mass, vigour, and dormancy [46]. However, although maternal effects may impose spatiotemporal impacts on the SMR of specific seed collections from different populations, these effects may underpin the variance around the species’ mean performance. Exploring these patterns within and between species is an obvious extension of our dataset, with a key objective of our study to establish a large comparative base for such future investigations.

The PGLS regression slope (0.75), which importantly accounts for phylogenetic as well as allometric patterns in SMR, is the best current estimate of the allometric slope for plant seeds, and calculation of species’ SMR residuals for comparative studies. The significantly higher PGLS SMR residuals of crop than native species (table 2) is not surprising as crop species are under intense artificial selection for agriculturally relevant characteristics such as uniform, rapid germination and high productivity [97]. Essentially, these crop plants have been bred to have seeds that are primed for rapid, highly energetic germination (associated with high SMR residuals).

We found no significant relationship between SMR residuals and maximum germination or germination speed (table 2). However, seed dormancy for some species may be masking a potential relationship between these traits. Although we used the best-known dormancy-alleviating treatments for species known to produce dormant seeds, some species nevertheless had low germination suggesting only a proportion of the entire sample had dormancy alleviated, resulting in a lower maximum germination and in turn slower germination speed than potentially possible (and also low SMR residuals). However, the patterns of SMR for dormant seeds also included species (e.g. *Solanum* spp.) with high SMR residuals, consistent with the active SMR of non-dormant seeds of other species, which nevertheless did not germinate. Dormancy is not a factor in crop species in our dataset, and their higher maximum germination (*G*_max_ = 87.8%) and shorter germination time (*T*_50_ = 3.17 days) than wild-collected, native and weed (66.1% and 8.27 days) species was associated with higher SMRs. Therefore, some measure of germination speed, particularly once dormancy has been fully accounted for, may contribute to our observed relationship between SMR residuals, climate of origin and whether seeds were wild or agriculturally sourced.

Our climatic analyses of PGLS residuals (native species only; table 2) showed the general pattern that seeds from warm, arid climates had higher SMR residuals compared to seeds from cooler, wetter environments. This pattern for seeds is opposite to the broad-scale patterns for ectothermic animals [98]. Several species in our study were from the Pilbara region of Western Australia (BWh climate region; electronic supplementary material, figure S2), which receives most of its rainfall as sporadic events often associated with tropical cyclones over the hot summer period [99]. These species produce seeds that germinate rapidly to take advantage of these transient rainfall events (time taken for 10% of the population to germinate *T*_10_ ≈ 2.2 days (*n* = 49 spp. [34,48]), as do seeds in arid zones worldwide [100]. A high seed MR might therefore be selected for in ecosystems with unpredictable rainfall and short periods of soil moisture availability. By comparison, seeds collected from the cooler, wetter, southwest Australian floristic region had lower SMR residuals (at 20°C). Species from this region produce seeds that germinate from late autumn through to late winter when temperatures are mild, rainfall is frequent and germination is slow, commonly requiring a period of many days or weeks to complete (*T*_10_ ≈ 8.7 days; *n* = 48 spp. [48,57]). Our analyses are restricted to three climate zones from across Western Australia which limits our capacity to extrapolate to eco-climatic correlates of seed MRs from a wide range of other climate types such as alpine environments and tropical forests. Expanding the dataset with species from other regions of the world will no doubt prove insightful in strengthening links between climate, MRs and seed traits more broadly.

Phylogenetically informed analyses have the potential to reduce or even remove effects of other physiological, ecological or environmental variables that also have a similar phylogenetic pattern as SMR. For example, a strong relationship between BMR and diet for phyllostomid bats by OLS was removed after accounting for phylogeny [101], because the diet category for bat lineages was completely confounded by phylogeny (i.e. all representatives of different phylogenetic groups had the same diets). Similarly, the value of making a ‘phylogenetic correction’ has been debated when examining ecological or other patterns (i.e. how to distinguish trait variation that is attributable to phylogeny versus ecology [102–104]). For our study, the insignificant difference between OLS and PGLS models, and the similarity of allometric slopes, suggests that we can confidently resolve ecological and environmental patterns from both OLS and PGLS SMR residuals. We also analysed the OLS residuals as we did for PGLS residuals and found no differences in any interpretations.

We have demonstrated here that the relationship between seed size and MR conforms to the fundamental allometric relationship found for most other plant and animal groups. Heavier seeds had a lower SMR per unit mass, which may be energetically rate-limiting in mobilizing stored resources for germination, essentially restricting potential growth or germination speeds. Germination success represents a key life-history stage that significantly shapes plant populations and community structures [105]. Here, we have established patterns of domestication and high SMR that are consistent with selection for rapid, consistently high germination success. Similarly, climatic drivers are well-established effectors of plant ecology, and we have established a directional link between the energetic state of the seed and climatic patterns, which suggests that SMR is a functional trait that underpins the ecology and evolution of seeds [106], and vegetative plants [107]. Future work is needed to focus on further factors that we consider might contribute further to species-specific variation in seed MRs. Other seed traits such as embryo type or size [108,109] that relate to the amount of metabolically active tissue contained within seeds, and the thickness and composition of the testa, including the presence or absence of mucilage, lignification and lipids can cause significant differences in gas exchange between species [110] and may contribute to the variation between species we observed. We also need to document the effect on SMR of seeds of ambient relative humidity, which influences seed water content, as has been done for many animal groups in terms of cryptobiosis (anhydrobiosis, osmobiosis, cryobiosis and anoxybiosis [111–113]). The metabolic ecology of seeds conforms to the expected mass-MR relationship established across a wide taxonomic range. We have, however, demonstrated new patterns in the ecological and evolutionary correlates of this relationship that are specific to plant seeds and suggest a rich field of research potential in the metabolic ecology of plants seeds.

## Data Availability

The data that support the findings of this study, and all analysis code, are included and available as the electronic supplementary material online [114].
